# Diagnostic Power of Cytokine M-CSF, Metalloproteinase 2 (MMP-2) and Tissue Inhibitor-2 (TIMP-2) in Cervical Cancer Patients Based on ROC Analysis

**DOI:** 10.1007/s12253-019-00626-z

**Published:** 2019-02-28

**Authors:** Emilia Lubowicka, Monika Zbucka-Kretowska, Iwona Sidorkiewicz, Monika Zajkowska, Ewa Gacuta, Andrzej Puchnarewicz, Lech Chrostek, Maciej Szmitkowski, Sławomir Ławicki

**Affiliations:** 1 Department of Esthetic Medicine, Medical University of Bialystok, Akademicka 3, 15-267 Bialystok, Poland; 2 Department of Reproduction and Gynecological Endocrinology, Medical University of Bialystok, 15-276 Bialystok, Poland; 3 Department of Biochemical Diagnostics, Medical University of Bialystok, 15-269 Bialystok, Poland; 4 Department of Perinatology, Medical University of Bialystok, 15-276 Bialystok, Poland; 5 Department of Urology, Provincial Hospital, 15-278 Bialystok, Poland; 6 Department of Population Medicine and Civilization Diseases Prevention, Medical University of Bialystok, 15-269 Bialystok, Poland

**Keywords:** M-CSF, MMP-2, TIMP-2, Cervical cancer, Tumor markers

## Abstract

Macrophage colony-stimulating factor (M-CSF), matrix metalloproteinase-2 (MMP-2) and its specific tissue inhibitor (TIMP-2) may play an important role in the pathogenesis of cancer disease. We investigated the plasma levels and diagnostic power (ROC curve analysis) of M-CSF, MMP-2, TIMP-2 and tumor markers CA 125 and SCC-Ag in cervical cancer (CC) patients as compared to control group. The study included 89 patients with cervical cancer. The control group consisted of 50 healthy, untreated women. The plasma levels of M-CSF, MMP-2 and TIMP-2 were determined using ELISA, CA 125 and SCC-Ag – by CMIA method. The median levels of M-CSF, TIMP-2, SCC-Ag and CA 125 in the entire group of CC were significantly different than compared to the healthy women group. MMP-2 showed the highest value of sensitivity from all examined parameters (in stage I of CC – 93.10%, II – 82.76%, III and IV – 96.88%, total group – 92.05%). The highest specificity was obtained by M-CSF (86%). The area under the ROC curve (AUC) of M-CSF (0.8051) was the largest of all the tested parameters (even higher than commonly used tumor markers) in the group of cervical cancer. The combination of M-CSF, MMP-2 or TIMP-2 with SCC antigen resulted in an increase AUCs in all cases (0.8760;0.7880;0.8081;respectively). The findings of this study suggest the usefulness of all examined parameters in the diagnostics of CC patients. Out of the tested substances, M-CSF also appears to be the best candidate for cancer diagnostics in all stages of the disease, based on ROC analysis.

## Introduction

Cancer of the uterine cervix is the leading cause of death among gynecological cancers in developing countries and the fourth principal cause of cancer-related death in women worldwide [1]. High-risk human papillomavirus (HPV) infection is considered the most important risk factor associated with the development of this tumor, and it is present in 99.7% of invasive cervical tumors worldwide including essentially all squamous cell cancers and adenocarcinomas [2].

Reliable methods for the accurate identification of both the presence and severity of cervical intraepithelial neoplasia (CIN) and extent of spread of invasive carcinomas of the cervix (IC) are critical. The primary objective of cervical cancer screening is to detect cervical intraepithelial neoplasia (CIN 3) sufficiently early so that it can be treated to prevent the development of cancer. Screening based on cytological testing (commonly known as the smear test or Pap test) is still the most important diagnostic technique for detecting pre-invasive cervical cancer [3]. Despite the prevention and early detection of cervical cancer, it is still the second leading cause of cancer- related death in young women worldwide. Further research is thus required to select prognostic biomarkers and therapeutic targets. No marker is completely specific, and therefore, diagnostic testing must be used in conjunction with morphological and clinical findings.

Macrophage colony-stimulating factor (M-CSF) is a hematopoietic growth factor that stimulates the proliferation and differentiation of monocytes to macrophages. In inflammation M-CSF induces macrophages to secrete cytokines and proteases, thereby enhancing the macrophages’ ability to combat microbial infections [4]. Increased expression of M-CSF and its receptor is correlated with poor prognosis in breast, ovarian and prostate cancer [5–11]. M-CSF has mainly been studied in breast carcinomas, where it is commonly expressed [12, 13]. Several other candidate markers have also been implicated, paricularly those involved in tumor invasion. A few studies have connected matrix metalloproteinases (MMPs), particularly gelatinases (MMP-2 and MMP-9), to tumor angiogenesis and growth [14]. MMP-2 and MMP-9 have been shown to be upregulated in angiogenic lesions [15]. The activity of the metalloproteinases is controlled by macroglobulins and, predominantly, tissue inhibitors of metalloproteinases (TIMPs). TIMP-2 functions include binding and inhibiting the proteolytic activity of MMPs and the activation of proMMP-2 [16]. Therefore, changes in TIMP-2 and MMP-2 levels would also determine whether TIMP-2 role is promoting or inhibitory. The main activation route of MMP-2 on the cell surface is by the formation of a molecular complex containing MMP-2, membrane type *1-*matrix metalloproteinase *1 (*MT1-MMP) and TIMP-2 [17]. Imbalance between the activity of MMPs and TIMPs has been attributed to the ability of cancer cells to migrate [18]. TIMP-2 correlates with poor prognosis in many types of cancer [19–21].

The aim of this study was to determine plasma levels of M-CSF, MMP-2 and TIMP-2 in comparison to CA 125 and SCC-Ag concentrations in patients with cervical cancer in relation to the control group (healthy subjects). Additionally, the diagnostic criteria: sensitivity (SE) and specificity (SP) were defined in the study. Furthermore, the study defined the receiver-operating characteristics (ROC) curve for all the tested parameters alone and in combination with tumor markers (CA 125 and SCC-Ag). The data obtained in the present study may be used in evaluating the usefulness (especially diagnostic power) of the tumor marker panel in the diagnostics of cervical cancer patients.

## Material and Methods

### Human Subjects

Table 1 shows the tested and control group. The study comprised 89 patients with invasive primary carcinoma of the uterine cervix who were referred to the Department of Gynecology, Bialystok Medical University Teaching Hospital, Poland, between 2012 and 2016. Clinical stages and histological classification based on the criteria of the International Federation of Gynecology and Obstetrics (FIGO) were established in all cases. Written consent including participants’ own statements regarding their medical history (i.e. data related to reproductive history, personal or family history of cancer, general health issues - hospitalization or surgery, use of medication) and lifestyle habits including smoking was obtained from all the subjects. None of the patients had received chemo- or radiotherapy before blood sample collection. Pretreatment staging procedures included physical and blood examinations, ultrasound scanning and chest X-rays. In addition, CT (computed tomography) scans or MRI (magnetic resonance imaging) were performed where necessary. The control group included 50 healthy and untreated women (aged 22–61 years). In these women cervical smears were examined by a gynecologist prior to blood collection. In addition, a reproductive organ ultrasound scan was performed where necessary. All subjects had undergone annual check-ups (laboratory tests, USG, chest x-ray, cervical cytology screening, mammography). The study was approved by the local Ethics Committee (R-I-002/239/2014) and all the patients gave their informed consent for study participation.

**Table 1:** Characteristics of cervical cancer patients and control group (healthy women)

Study group			Number of patients
Tested Group	Cervical cancer patients	Squamous cell carcinoma	89
	Median age (range)		47 (25–61)
	Tumor stage	I	29
		II	28
		III and IV	32
	Menopausal status		
	- premenopausal		69
	- postmenopausal		20
Control group	Healthy women		
	Median age (range)		50
	Menopausal status:		42 (22–61)
	- premenopausal		39
	- postmenopausal		11

### Plasma Collection and Storage

Venous blood samples were collected from each patient. Blood was collected into heparin sodium tubes, centrifuged 3500 rpm for 20 min to obtain plasma samples, and stored at −85 °C until assayed.

### Measurement of M-CSF, MMP-2, TIMP-2, CA 125 and SCC-Ag

The examined parameters (MMP-2, TIMP-2, and M-CSF) were measured with enzyme-linked immunosorbent assay (ELISA) (Quantikine Human M-CSF Immunoassay; R&D systems, Abingdon, United Kingdom), according to the manufacturer’s instructions. This assay employs the quantitative sandwich enzyme immunoassay technique. Plasma concentrations of SCC-Ag and CA 125 were measured by chemiluminescent microparticle immunoassay (CMIA) (Abbott, Chicago, IL, USA). The intra-assay coefficient of variation (CV%) of CA 125 is reported to be 2.4% at a mean concentration of 43.50 U/mL, SD = 1.1. SCC-Ag is reported to be 4.3% at a mean concentration of 1.97 ng/mL, SD = 0.085. M-CSF is reported to be 3.4% at a mean concentration of 227 pg/mL, SD = 7.7. MMP-2 is reported to be 3.8% at a mean concentration of 11.20 ng/mL, SD = 0.42, TIMP-2 to be 6.0% at a mean concentration of 2.90 ng/mL, SD = 0.173. The inter-assay coefficient of variation (CV%) of CA 125 is reported to be 3.9% at a mean concentration of 43.50 U/ml, SD = 1.7. SCC-Ag is reported to be 5.1% at a mean concentration of 1.97 ng/mL, SD = 0.1. M-CSF to be 3.1% at a mean concentration of 232 pg/ml, SD = 7.3. MMP-2 is reported to be 6.6% at a mean concentration of 11.10 ng/mL, SD = 0.738, TIMP-2 to be 6.7% at a mean concentration of 2.79 ng/mL, SD = 0.188. The value of intra- and inter-assay CVs were calculated by the manufacturers and enclosed in the reagent kits. The assay does not exhibit cross-reactivity or interference with numerous human cytokines and other growth factors. Duplicate samples were assessed for each patient.

### Statistical Analysis

Statistical analysis was performed using STATISTICA 12.0 PL (StatSoft, Tulsa, OK, USA). Preliminary statistical analysis (Chi-square test) revealed that the tested parameters and tumor marker levels did not follow a normal distribution. Consequently, the Mann–Whitney U test was used for statistical analysis between cancer and control group. Additionally, statistical analysis between groups with different stages of CC was performed using the Kruskal–Wallis test, and a multivariate analysis of various data - with the post-hoc Dwass Steele–Crichlow–Flinger test. Statistically significant differences were defined as comparisons resulting in *p* < 0.05. Diagnostic sensitivity (SE) and specificity (SP) were calculated. The *cut off* values were calculated by Youden’s index (as a criterion for selecting the optimum cut-off point) and for each of the tested parameters was as follows: M-CSF – 394.42 pg/mL; MMP-2 – 155.92 ng/mL; TIMP-2 – 82.12 ng/mL; CA 125–13.44 U/mL; SCC-Ag – 0.89 ng/mL. In the analyses of both diagnostic performance (SE, SP) and ROC curve, only healthy subjects were used as a control group. The construction of the ROC curves was performed using the GraphRoc program for Windows (Windows,Royal, AR, USA) and the areas under the ROC curve (AUC) were calculated.

## Results

Table 2 presents the median and the range of plasma levels of the investigated parameters and CA 125 and SCC-Ag in the tested groups. The median values for M-CSF (510.55 pg/mL), similar to those of the commonly accepted tumor markers CA 125 (17.99 U/mL) and SCC-Ag (1.20 U/mL), in the entire group of CC were significantly higher compared with the values in healthy subjects (251.50 pg/mL; 11.70 U/mL,0.75 U/mL, respectively) (*p* < 0.05). Additionally, the median value of TIMP-2 (76.00 ng/mL) in the total cervical cancer group was significantly lower compared with the values in healthy subjects (87.25 ng/mL) (*p* < 0.05).

**Table 2:** Plasma levels of tested parameters and CA 125 and SCC-Ag in patients with cervical cancer and in control group

Groups tested	M-CSF (pg/mL)	MMP-2 (ng/mL)	TIMP-2 (ng/mL)	SCC-Ag (U/mL)	CA 125 (U/mL)
Cervical cancer (median, range)					
Stage I	422.55^a^ (102.15–2513.75)	200.00 (124.84–352.00)	70.00^a^ (40.00–160.00)	1.29 (0.38–2.20)	14.40 (6.60–49.60)
Stage II	510.55^a^ (95.26–1304.80)	218.00 (129.80–379.00)	71.50 (26.93–120.00)	1.20 (0.45–5.90)	17.40^a^ (4.40–77.41)
Stages III and IV	578.50^a^ (113.05–2511.95)	221.00^a^ (140.50–351.96)	84.60^b/c^ (50.00–156.00)	1.20 (0.30–14.10)	25.65 ^a/b^ (6.34–120.10)
Total group	510.55^a^ (95.23–2513.75)	214.00 (124.84–379.00)	76.00^a^ (26.93–160.00)	1.20 ^a^ (0.30–14.10)	17.99 ^a^ (4.40–120.10)
Control groups (median, range)					
Healthy women	251.50 (119.63–935.29)	202.95 (24.30–397.20)	87.25 (42.50–132.50)	0.75 (0.40–1.60)	11.70 (3.50–36.60)

^a^ Statistically significant when patients with CC compared with healthy women.

^b^ Statistically significant when patients with CC stages III and IV compared with patients with CC stage I

^c^ Statistically significant when patients with CC stages III and IV compared with patients with CC stage II

Similarly, we observed statistically significantly higher concentrations of M-CSF in all the analyzed groups in relation to CC stage compared with healthy women (*p* < 0.05 in all cases). Additionally, we observed significantly higher concentrations of CA 125 in all the analyzed groups in all stages of cervical cancer (with the exception of stage I) in comparison with healthy women. Significantly higher concentrations of MMP-2 in stages III and IV were also observed (*p* < 0.05). Furthermore, significantly lower concentrations of TIMP-2 in stage I were also noticed (*p* < 0.05).

We also observed significantly higher concentrations of TIMP-2 in stages III and IV of cancer compared with stage I or II (TIMP-2: I vs. III and IV *p* < 0.05 and II vs. III and IV *p* < 0.05). Furthermore, we detected significantly higher plasma levels of CA 125 when stages III and IV was compared to stage I (*p* < 0.05).

Diagnostic criteria for tumor markers are sensitivity (SE) and specificity (SP) (Table 3). We indicated that the SE of the tested cytokines in the total cancer group was the highest for MMP-2 (92.05%). The combined use of M-CSF, MMP-2 or TIMP-2 with the commonly accepted tumor markers (antigen SCC and CA 125) resulted in an increase in the sensitivity range in the total CC group. Maximum diagnostic sensitivity (98.88%) was obtained for the combination of MMP-2 with CA 125. Among all parameters, the highest SE in all stages of cancer was observed for MMP-2 (in stage I of CC – 93.10%, in stage II of CC – 82.76%, and in stages III and IV of CC – 96.88%). The combined use of the tested parameters and CA 125 and SCC-Ag resulted in an increase of SE in every stage of CC. A maximum range was observed for the combination of MMP-2 with CA 125 in stages II, III and IV (100% in all cases).

**Table 3:** Diagnostic criteria of tested parameters and in combined analysis with CA 125 and SCC-Ag in cervical cancer patients

Tested parameters	Diagnostic criteria (%)	Cervical cancer			
		Stage I	Stage II	Stages III and IV	Total
M-CSF	SE	51.72	75.00	78.13	69.41
	SP	86.00	86.00	86.00	86.00
MMP-2	SE	93.10	82.76	96.88	92.05
	SP	58.00	58.00	58.00	58.00
TIMP-2	SE	17.24	17.86	59.38	32.18
	SP	60.00	60.00	60.00	60.00
CA 125	SE	62.07	82.14	96.88	80.00
	SP	68.00	68.00	68.00	68.00
SCC-Ag	SE	75.86	78.57	78.13	81.18
	SP	74.00	74.00	74.00	74.00
M-CSF + CA 125	SE	79.31	93.10	100.00	91.76
	SP	66.00	66.00	66.00	66.00
MMP-2 + CA 125	SE	96.55	100.00	100.00	98.88
	SP	30.00	30.00	30.00	30.00
TIMP-2 + CA 125	SE	68.97	85.71	100.00	85.39
	SP	28.00	28.00	28.00	28.00
M-CSF + SCC-Ag	SE	86.21	92.86	93.75	91.76
	SP	66.00	66.00	66.00	66.00
MMP-2 + SCC-Ag	SE	96.55	96.43	100.00	96.63
	SP	36.00	36.00	36.00	36.00
TIMP-2 + SCC-Ag	SE	75.86	79.31	87.50	82.02
	SP	30.00	30.00	30.00	30.00

Abbreviations: *CA*, cancer antigen; *MMP-2*, matrix metalloproteinase-2; *TIMP-2*, tissue inhibitor of metalloproteinase-2; *M-CSF*, macrophage-colony stimulating factor; *SE*, sensitivity; *SP*, Specificity

The diagnostic SP of the tested parameters was the highest for M-CSF (86%) and was higher than that for CA 125 (68%) and SCC-Ag (74%).

**Fig. 1:**
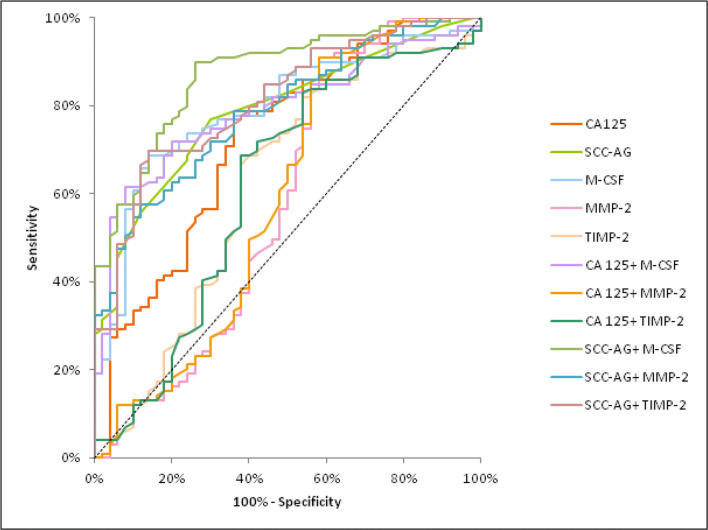
Diagnostic criteria of ROC curve for examined parameters in combination with CA 125 and SCC-Ag in the cervical cancer group. Abbreviations: ROC, receiver-operating characteristics; M-CSF, macrophage-colony stimulating factor; MMP-2, matrix metalloproteinase-2; TIMP-2, tissue inhibitor of metalloproteinase-2

The relationship between the diagnostic SE and SP is illustrated by the ROC curve (Tables 4 and 5). The AUC indicates the clinical usefulness of a tumor marker and its diagnostic power. We noticed that the AUC for M-CSF (0.8051) in the total CC group was larger than the area of SCC-Ag (0.7866), CA 125 (0.7340), TIMP-2 (0.6186) and MMP-2 (0.5882). Moreover, areas under the ROC curve for M-CSF and TIMP-2, similarly as for CA 125 and SCC-Ag, were statistically significantly larger in comparison to AUC = 0.5 (borderline of the diagnostic usefulness of the test) (*p* < 0.001; *p* = 0.0257; *p* < 0.001; *p* < 0.001; respectively) The combined analysis of AUC for M-CSF, MMP-2, and TIMP-2 with antigen SCC resulted in an increase in the areas in all cases (0.8760; 0.7880; 0.8081; respectively) (Fig. 1).

**Table 4:** Diagnostic criteria of ROC curve for tested parameters and CA 125 and SCC-Ag in total group of CC

Tested parameters	AUC	SE	95% C.I. (AUC)	*p* (AUC = 0.5)
ROC criteria in cervical cancer (total)				
M-CSF	0.8051	0.0383	0.730–0.880	<0.001
MMP-2	0.5882	0.0566	0.477–0.699	0.1195
TIMP-2	0.6186	0.0532	0.514–0.723	0.0257
CA 125	0.7340	0.0461	0.644–0.824	<0.001
SCC-Ag	0.7866	0.0383	0.711–0.862	<0.001
M-CSF + CA 125	0.8006	0.0376	0.727–0.874	<0.001
MMP-2 + CA 125	0.5963	0.0563	0.486–0.707	0.0874
TIMP-2 + CA 125	0.6166	0.0537	0.511–0.722	0.0299
M-SCF + SCC-Ag	0.8760	0.0296	0.818–0.934	<0.001
MMP-2 + SCC-Ag	0.7880	0.0381	0.713–0.863	<0.001
TIMP-2 + SCC-Ag	0.8081	0.0370	0.736–0.881	<0.001

*p* - statistically significantly larger AUCs compared to AUC = 0.5

Abbreviations: *ROC*, receiver-operating characteristics; *CA*, cancer antigen; *AUC*, area under the ROC curve; *SE*, standard error; *CI*, confidence interval; *M-CSF*, macrophage-colony stimulating

factor; *MMP-2*, matrix metalloproteinase-2; *TIMP-2*, tissue inhibitor of metalloproteinase-2

**Table 5:** Diagnostic criteria of ROC curve for tested parameters and CA 125 and SCC-Ag in all stages of CC

Tested parameters	AUC	SE	95% C.I. (AUC)	*p* (AUC = 0.5)
ROC criteria in cervical cancer (I stage)				
M-CSF	0.7109	0.0631	0.582–0.829	0.0011
MMP-2	0.5484	0.0658	0.421–0.678	0.4223
TIMP-2	0.6747	0.0622	0.562–0.799	0.0064
CA 125	0.6429	0.0614	0.522–0.760	0.0188
SCC-Ag	0.8022	0.0521	0.709–0.908	<0.001
M-CSF + CA 125	0.8001	0.0641	0.591–0.839	<0.001
MMP-2 + CA 125	0.5499	0.0649	0.423–0.677	0.4646
TIMP-2 + CA 125	0.6661	0.0627	0.551–0.796	0.0088
M-SCF + SCC-Ag	0.8498	0.0440	0.768–0.935	<0.001
MMP-2 + SCC-Ag	0.7824	0.0534	0.684–0.891	<0.001
TIMP-2 + SCC-Ag	0.8320	0.0461	0.758–0.932	<0.001
ROC criteria in cervical cancer (II stage)				
M-CSF	0.8042	0.0532	0.698–0.912	<0.001
MMP-2	0.5564	0.0639	0.433–0.679	0.3915
TIMP-2	0.6898	0.0611	0.558–0.799	0.0062
CA 125	0.7297	0.0571	0.604–0.836	<0.001
SCC-Ag	0.7973	0.0523	0.691–0.908	<0.001
M-CSF + CA 125	0.8002	0.0559	0.678–0.910	<0.001
MMP-2 + CA 125	0.5647	0.0634	0.441–0.688	0.3082
TIMP-2 + CA 125	0.6662	0.0612	0.551–0.782	0.0054
M-SCF + SCC-Ag	0.8857	0.0410	0.809–0.968	<0.001
MMP-2 + SCC-Ag	0.7931	0.0529	0.698–0.901	<0.001
TIMP-2 + SCC-Ag	0.8891	0.0458	0.748–0.930	<0.001
ROC criteria in cervical cancer (III and IV stages)				
M-CSF	0.8797	0.0428	0.796–0.951	<0.001
MMP-2	0.6588	0.0601	0.538–0.776	0.0098
TIMP-2	0.5107	0.0668	0.388–0.639	0.8124
CA 125	0.8114	0.0498	0.717–0.901	<0.001
SCC-Ag	0.7616	0.0564	0.652–0.879	<0.001
M-CSF + CA 125	0.8702	0.0443	0.781–0.952	<0.001
MMP-2 + CA 125	0.6698	0.0592	0.550–0.781	0.0039
TIMP-2 + CA 125	0.5201	0.0649	0.398–0.649	0.7505
M-SCF + SCC-Ag	0.8885	0.0386	0.818–0.962	<0.001
MMP-2 + SCC-Ag	0.7881	0.0515	0.687–0.891	<0.001
TIMP-2 + SCC-Ag	0.7607	0.0543	0.660–0.869	<0.001

*p* - statistically significantly larger AUCs compared to AUC = 0.5

Abbreviations: *ROC*, receiver-operating characteristics; *CA*, cancer antigen; *AUC*, area under the ROC curve; *SE*, standard error; *CI*, confidence interval; *M-CSF*, macrophage-colony stimulating

factor; *MMP-2*, matrix metalloproteinase-2; *TIMP-2*, tissue inhibitor of metalloproteinase-2

**Fig. 2:**
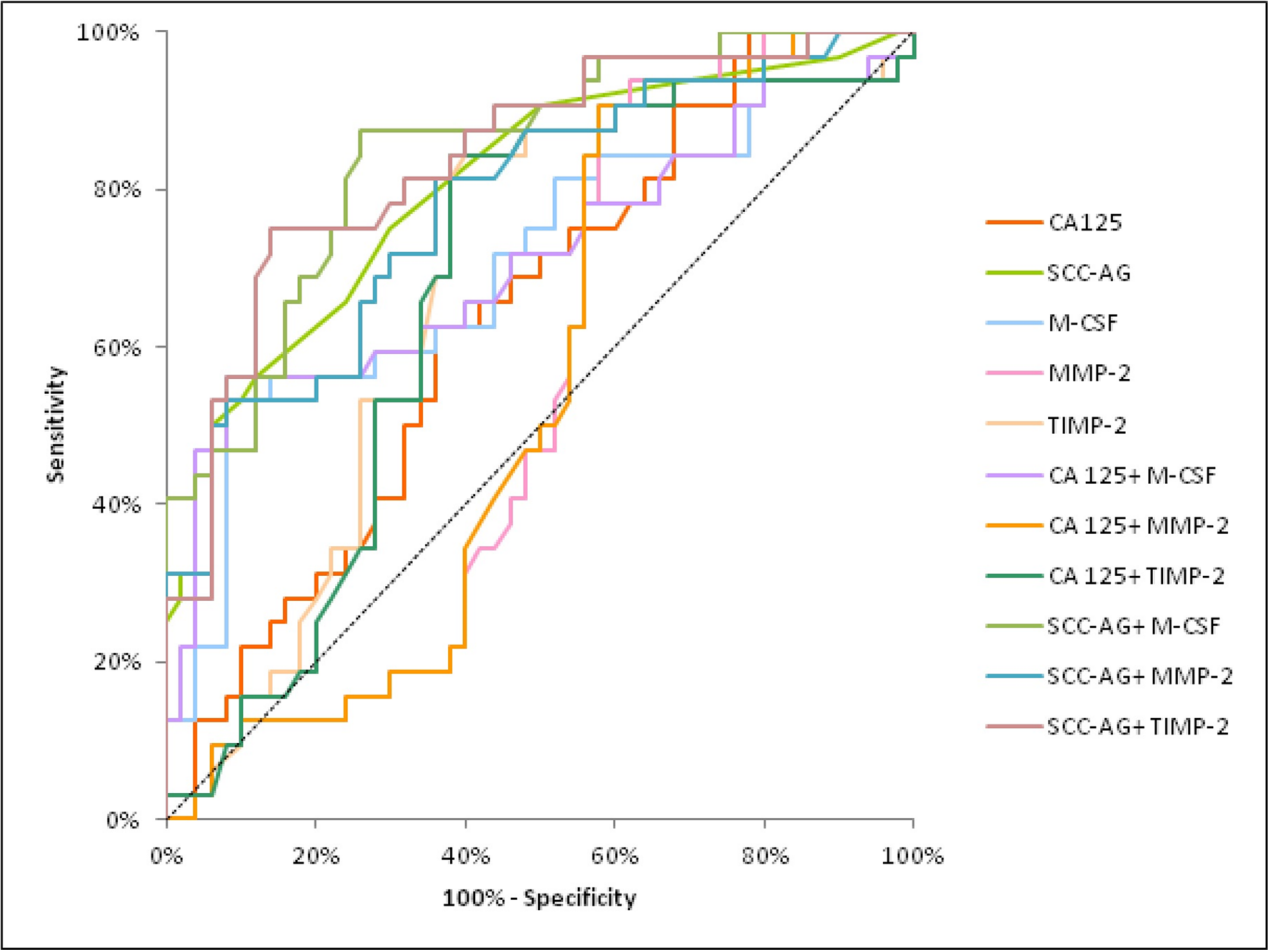
Diagnostic criteria of ROC curve for examined parameters in combination with CA 125 and SCC-Ag in stage I of cervical cancer group. Abbreviations: ROC, receiver-operating characteristics; M-CSF, macrophage-colony stimulating factor; MMP-2, matrix metalloproteinase-2; TIMP-2, tissue inhibitor of metalloproteinase-2

**Fig. 3:**
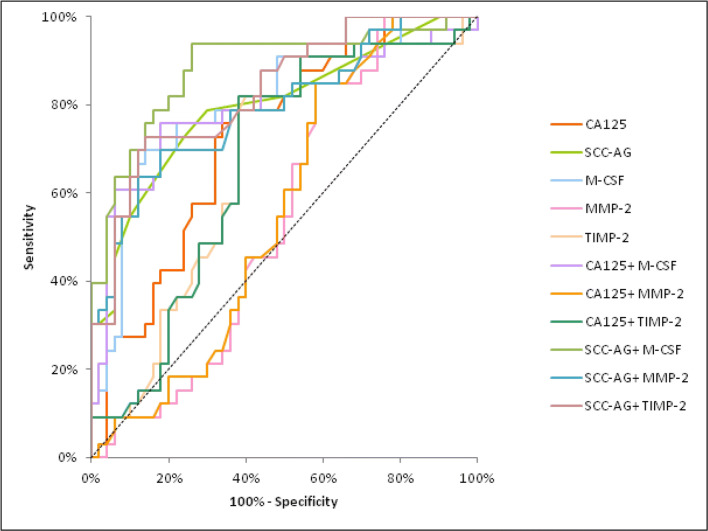
Diagnostic criteria of ROC curve for examined parameters in combination with CA 125 and SCC-Ag in stage II of cervical cancer group. Abbreviations: ROC, receiver-operating characteristics; M-CSF, macrophage-colony stimulating factor; MMP-2, matrix metalloproteinase-2; TIMP-2, tissue inhibitor of metalloproteinase-2

**Fig. 4:**
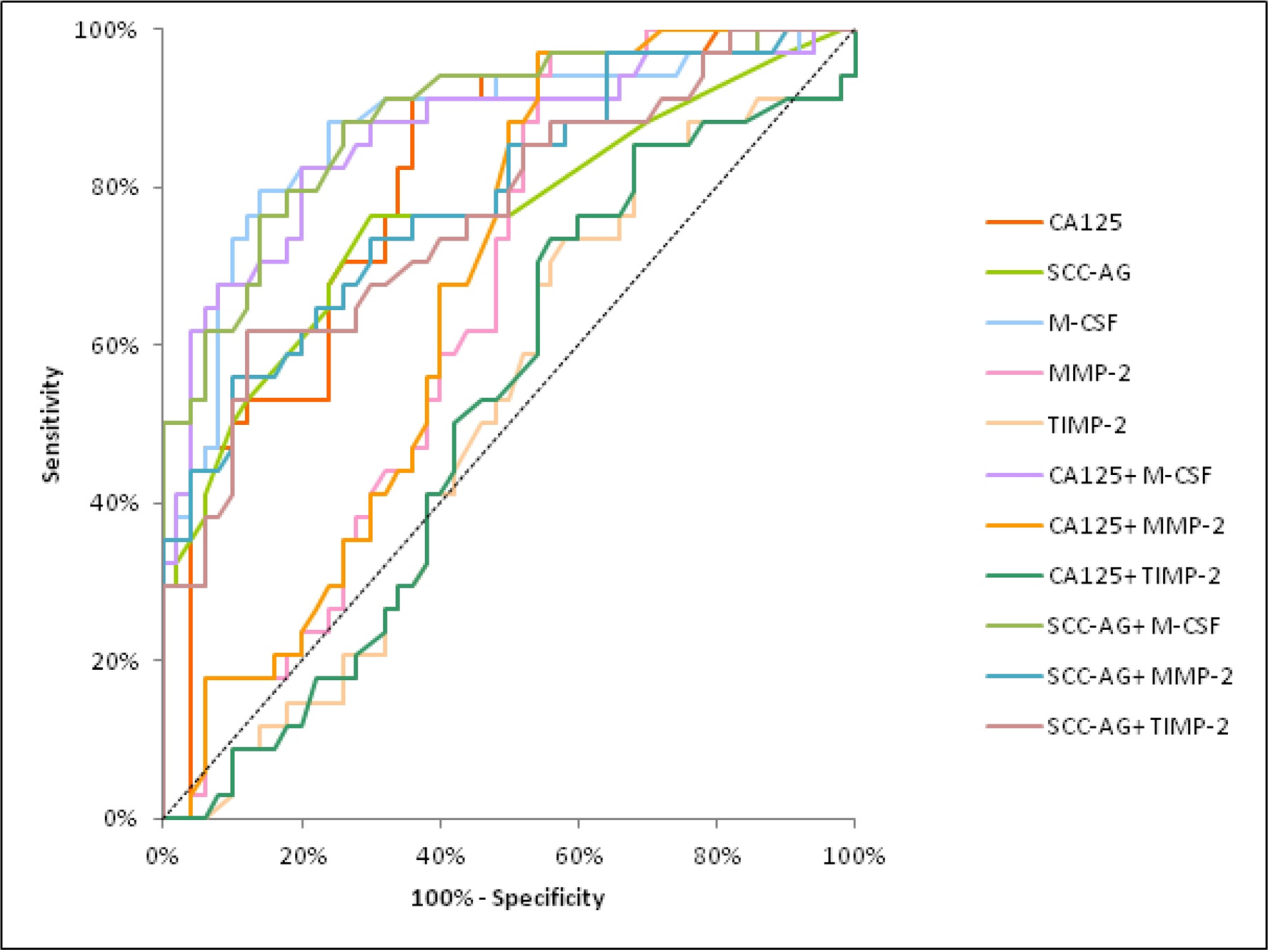
Diagnostic criteria of ROC curve for examined parameters in combination with CA 125 and SCC-Ag in stages III and IV of cervical cancer group. Abbreviations: ROC, receiver-operating characteristics; M-CSF, macrophage-colony stimulating factor; MMP-2, matrix metalloproteinase-2; TIMP-2, tissue inhibitor of metalloproteinase-2

The AUC of M-CSF and MMP-2 demonstrated a distinct increase, concomitant with CC stage, identically to CA 125. In stage I of CC the highest AUC of all the tested parameters was found in SCC-Ag (0.8022) and it was the parameter which was statistically significantly larger in comparison to AUC = 0.5 (*p* < 0.001), similarly to M-CSF (*p* = 0.0011), TIMP-2 (*p* = 0.0064) and CA 125 (*p* = 0.0188) (Fig. 2). In stage II of CC the highest AUC of all the tested parameters was observed in M-CSF (0.8042; *p* < 0.001) and it was marginally higher than SCC-Ag (0.7973). Moreover, the AUCs for M-CSF and TIMP-2, similarly to those for CA 125 and SCC-Ag, were statistically significantly larger in comparison to AUC =0.5 (*p* < 0.001; *p* = 0.0062; *p* < 0.001; *p* < 0.001; respectively) (Fig. 3). In stages III and IV of CC the highest AUC of all the tested parameters was observed in M-CSF (0.8797; *p* < 0.001) and it was marginally higher than CA 125 (0.8114; *p* < 0.001). Additionally, the AUCs for M-CSF and MMP-2, similarly as for CA 125 and SCC-Ag, were statistically significantly larger in comparison to AUC =0.5 (*p* < 0.001; *p* = 0.0098; *p* < 0.001; *p* < 0.001; respectively). (Fig. 4). The combined analysis of AUC for the tested parameters (M-CSF, MMP-2 or TIMP-2) with antigen SCC resulted in an increase in the areas in all stages of CC (*p* < 0.001).

## Discussion

The most promising markers appear to be serum cytokines, matrix metalloproteinases and their tissue inhibitors produced by a wide variety of cells, which play a central role in the regulation of inflammatory processes and the immune system. An ideal tumor marker should have a high sensitivity and specificity in order to discriminate between patients with cancer and those with benign conditions or healthy controls, and should also provide information related to tumor burden and activity [22]. Many new tumor markers have been discovered since the development of monoclonal antibodies, and the majority of tumors are now detected using them. Cytokines play an important role in tumor-stroma interaction, thus facilitating tumor progression and aggressiveness. Recombinant human M-CSF induces angiogenesis through macrophages by promoting VEGFA expression [23]. We have previously found increased concentrations of M-CSF in the plasma of patients with endometrial [24], breast [25] and ovarian cancer [26]. Moreover, M-CSF is a good candidate as a marker of colorectal [27] and gastric cancer [28]. A number of other authors have also pointed to the role of MMP-2 in cancer invasion and metastasis [29, 30]. Matrix metalloproteinases are able to remodel the extracellular matrix (ECM). It has been indicated that this enzymes might be produced by tumor cells [31]. The activity of MMP-2 is mainly controlled by interactions with its natural inhibitor – tissue inhibitor of metalloproteinase-2 (TIMP-2). The imbalance between these MMPs and TIMPs as a result of the increased production and activation of MMPs is responsible for cancer metastasis [31].

In the present study, the ELISA method was used to measure the plasma concentrations of M-CSF, MMP-2, TIMP-2 in cervical cancer patients. The levels of M-CSF, CA 125 and SCC-Ag were significantly higher compared to healthy subjects. Only the plasma levels of TIMP-2 were statistically significantly lower when compared to healthy controls. These findings are in agreement with our previous study showing that the median levels of M-CSF in cervical cancer patients are higher (510.55 pg/mL) than in healthy subjects (290.82 pg/mL) [32]. Our observation is supported by data regarding M-CSF from a previous report by Ławicki et al. [33] in breast cancer patients. They are also in line with the observations of other authors studying M-CSF levels in patients with pancreatic cancer [34]. Furthermore, it has observed that M-CSF concentrations are statistically different in every group (the analysis related to CC stage) compared to healthy subjects. Similar results were obtained by Ławicki et al. [35] who observed higher concentrations of M-CSF in all the analysed groups in relation to breast cancer stage compared to healthy women. Significantly elevated levels of M-CSF in advanced stages of cancer have also been found in patients with malignancies of the reproductive organs [8]. In our study, similar data were obtained for CA 125 (with the exception of stage I). Furthermore, elevated levels of M-CSF and CA 125 correspond to the positive correlations of M-CSF and CA 125 with more advanced stages of cervical cancer. Additionally, this study found high levels of MMP-2 in stages III and IV. The same observations regarding MMP-2 were reported by Ghosh et al. [36] who demonstrated that MMP-2 protein expression increased differentially in cervical cancer and it was associated with carcinoma stage.

In our study, the sensitivity of MMP-2 was the highest out of all the tested parameters (92.05%). Ghosh et al. [36] also showed high sensitivity values of MMP-2 in cervical cancer patients, although that study employed zymographic analysis to measure MMP-2 in the samples of cervical tissue. In the present study, the combined use of M-CSF, MMP-2 or TIMP-2 with the commonly accepted tumor markers resulted in an increase in SE values. Among all the parameters, the highest SE in all stages of cancer was observed for MMP-2 (93.10%, 82.76%; 96.88%; respectively). In the publication by Ławicki et al. [33] concerning patients with breast cancer, the sensitivity of MMP-2 was lower than that reported in the present study. The highest values of SE were obtained for the combination of MMP-2 with the commonly accepted tumor markers in all stages of cancer. In our previous study, the highest values were observed for the combination of M-CSF, TIMP-2 and CA 15–3 in all stages of breast cancer group [33].

In this study, the SP of M-CSF (86%) was the highest out of all the studied parameters and was higher than that of SCC-Ag (74%) and CA 125 (68%). In the case of M-CSF, the specificity reported in the paper by Ławicki et al. [37] on cervical cancer was marginally higher than that obtained in our study (92%). M-CSF specificity obtained by Vasiliades [34] et al. in the study on pancreatic cancer was lower than ours (62.5%). This discrepancy is probably due to the differences in the types of cancers studied.

The area under the ROC curve indicates the clinical usefulness of a tumor marker. In this study, the area under the ROC curve of M-CSF was the largest out of all the tested parameters in the total CC group. Additionally, we observed statistically significantly larger AUCs for the tested parameters (with the exception of MMP-2), compared to AUC = 0.5 (borderline of diagnostic usefulness of the test). The combined analysis of AUC for all the tested parameters with the commonly accepted tumor marker (SCC-Ag) resulted in an increase in the areas in all cases. A study by Ławicki et al. [32] reported the area under the ROC curve of M-CSF to be marginally lower than ours. Our results show that the diagnostic power (AUC) of the tested parameters, especially M-CSF, in the group of CC patients was marginally lower in comparison with the diagnostic power of M-CSF in the course of pancreatic cancer study conducted by Vasiliades et al. [34]. Moreover, the area under the ROC curve of M-CSF and the combined analysis of M-CSF with CA 125 and SCC-Ag were larger than the area of MMP-2 or TIMP-2 in all stages of cancer. In our previous study, we observed that M-CSF had the largest AUC in stage IV of breast cancer [35].

## Conclusions

The findings of this study suggest the usefulness of M-CSF, MMP-2 and TIMP-2 in the diagnostics of CC patients, particularly in combination with CA 125 and SCC-Ag. The area under the ROC curve was highest for the combination of M-CSF and the commonly accepted tumor markers, which indicates potential clinical significance of plasma M-CSF in the diagnosis of CC. Out of the tested substances, M-CSF also appears to be the best candidate for cancer diagnostics in all stages of the disease, based on ROC analysis.

## Abbreviations

CC: cervical cancer
M-CSF: macrophage colony-stimulating factor
MMP-2: matrix metalloproteinase-2
TIMP-2: tissue inhibitor of metalloproteinase-2
